# Ultrasonographic evaluation of cecal appendix diameter in pediatric population

**DOI:** 10.31744/einstein_journal/2022AO6935

**Published:** 2022-06-28

**Authors:** Marcos Roberto Gomes de Queiroz, Miguel José Francisco, Antonio Rahal, Victor Arantes Jabour, Guilherme Neves Lourenço Andrade, Paulo Savoia Dias da Silva, Rodrigo Gobbo Garcia, Marcelo dos Santos Pereira, Marina Ramos Santos, Pedro Andrade Alencar Luna, Yoshino Tamaki Sameshima, Fabiana Gual, Marcelo Guimarães Dutra, Beatriz Placca Germino, Isabella Ferreira Alves

**Affiliations:** 1 Hospital Israelita Albert Einstein São Paulo SP Brazil Hospital Israelita Albert Einstein, São Paulo, SP, Brazil.; 2 Hospital das Clínicas Faculdade de Medicina Universidade de São Paulo São Paulo SP Brazil Hospital das Clínicas, Faculdade de Medicina, Universidade de São Paulo, São Paulo, SP, Brazil.; 3 Faculdade Israelita de Ciências da Saúde Albert Einstein Hospital Israelita Albert Einstein São Paulo SP Brazil Faculdade Israelita de Ciências da Saúde Albert Einstein, Hospital Israelita Albert Einstein, São Paulo, SP, Brazil.

**Keywords:** Appendicitis/diagnostic imaging, Appendix, Ultrasonography, Child

## Abstract

**Objective:**

To stratify ultrasound samples in a pediatric population undergoing evaluation for acute appendicitis to examine the variability in cecal appendix diameter, in different age groups, and to determine whether there is a prevalent value for each age group.

**Methods:**

A retrospective cross-sectional study with 196 children aged 0 to 15 years. Data were extracted from reports of ultrasound examinations carried out between 2008 and 2015. Children with sonographic diagnosis of appendicitis or other signs of periappendiceal inflammation were excluded.

**Results:**

The evaluation of the anteroposterior measurement of the cecal appendix revealed a mean diameter of 4.14mm (standard deviation: 0.93mm; 95%CI: 3.86-4.14). Cecal appendix diameter did not differ significant between age groups.

**Conclusion:**

Evaluation of the anteroposterior diameter of the cecal appendix in centimeters in a sample of 196 children aged 0 to15 years revealed a mean diameter of 4.14mm (standard deviation, 0.93mm. There were no significant differences in cecal appendix diameter following stratification by age. Results indicate a single value can be adopted for mean cecal appendix diameter in pediatric populations.

## INTRODUCTION

Acute appendicitis is one of the most common disorders affecting the cecal appendix, especially in pediatric patients. Acute appendicitis is also a major cause of abdominal pain requiring surgical treatment seen in emergency rooms.^(1)^ Early detection by ultrasound (US) examination may prevent complications such as appendix perforation, abscess formation and sepsis, with positive impacts on perioperative morbidity and mortality.

Ultrasonography is one of the main diagnostic tools for initial assessment of abdominal pain and is the imaging modality of choice in patients with abdominal pain seen in the Emergency Department, according to the vast majority of related articles. It is particularly indicated for differential diagnosis of appendicitis due to short turnaround time, widely available, high repeatability and low cost. Also, the fact that it does not involve radiation makes it as an ideal modality for acute appendicitis diagnosis in pediatric populations.^(2)^

Sonographic diagnosis of acute appendicitis is based on direct (cecal appendix diameter, compressibility and loss of normal echotexture) and indirect (lymph node enlargement, locoregional hyperechogenicity, free abdominal fluid and reactive small bowel dilatation findings. The cross sectional diameter of the cecal appendix is one of the most important sonographic parameters for acute appendicitis diagnosis.^(3,4)^

In pediatric populations, most normality parameters for US measurement of abdominal structures are stratified by age,^(5)^ which may confuse the examiner during cecal appendix assessment in children. The establishment of normal reference values of appendix diameter for pediatric patients may be an important tool for recognition of potential sonographic abnormalities.

Several studies have been carried out in order to investigate the performance of US examination in the diagnosis of acute appendicitis. In a study by Binkovitz et al. with a population of 790 children aged under 18 years, US examination achieved 96% accuracy, 94.8% sensitivity and 96.3% specificity.^(6)^

Prompt, accurate sonographic diagnosis may also decrease the need for tomographic assessment and prevent negative laparotomies. In a multicenter cohort study carried out by Mittal et al. with 2,625 children aged 3 to 18 years suspected of acute appendicitis, the appendix could be clearly seen in US images with 88.8% accuracy, 72.5% sensitivity and 97% specificity.^(7)^ Therefore, there is strong evidence that US is an effective imaging modality for acute appendicitis diagnosis in children.^(8,9)^

However, consistent data regarding reference ranges for normal cecal appendix diameter in the general and particularly in the pediatric population are lacking. Hence the need to investigate this topic.

The establishment of reference ranges for normal cecal appendix diameter in the pediatric population may assist sonographic diagnostic assessment of abdominal pain in children with suspected acute appendicitis.

Unprecedented advancements in US equipment in last decades enabled imaging assessment of hollow abdominal viscera, from the gastroesophageal junction to the stomach, the small and large intestines and the anal canal. In this scenario, confirmation of normal cecal appendix diameter became the goal of routine US examination in all patients, regardless of age group. However, this is particularly important in pediatric patients, given the significance of this finding for the diagnosis of acute appendicitis and related conditions, which should be routinely described in sonographic reports.

## OBJECTIVE

To stratify sonographic measurements by age in a pediatric population investigated for acute appendicitis in order to examine the variability in normal cecal appendix diameter in different age groups and to determine whether there is a prevalent value for age.

## METHODS

Retrospective cross-sectional study with 196 children aged 0 to 15 years, based on data extracted from reports of US examinations (IU22, Philips Ultrasound Inc, Bothell, WA, USA) carried out between 2008 and 2015.

This study was approved and exempt from informed consent by the institutional Ethics Committee (protocol # 3.211.129, CAAE: 01863918.0.0000.0071).

**Figure 1:**
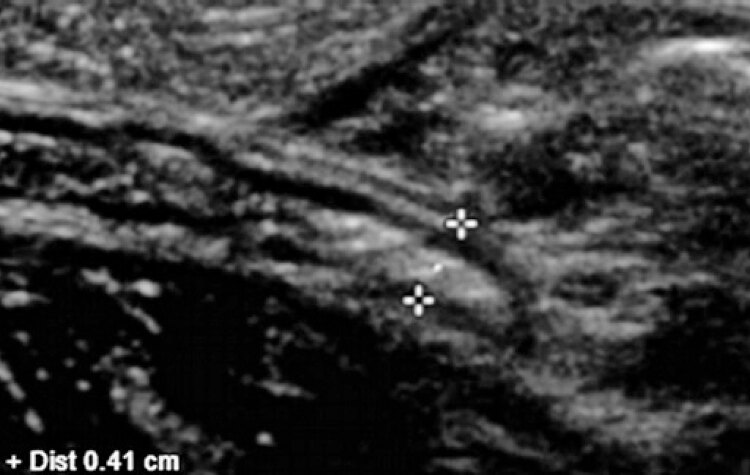
Normal cecal appendix - ultrasound measurement of cecal appendix diameter (longitudinal section)

**Figure 2:**
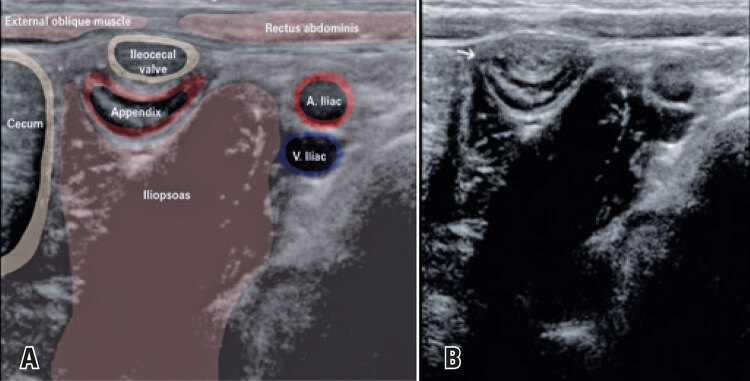
Sonographic images of abdominal structures. (A) Normal cecal appendix in the right iliac fossa (longitudinal section); (B) Typical blind end of this bowel- white arrow

Children with a sonographic diagnosis of appendicitis or other signs of periappendiceal inflammation, such as free abdominal fluid, adjacent fat blurring and lymph node enlargement were excluded. Settings used for US assessment of abdominal anatomy and respective layers are shown in the images below (Figures 1 and 2).

### Sample calculation

Based on clinical practice, the estimated cecal appendix diameter ranges from 0.17cm to 0.62cm (minimum and maximum value respectively), resulting in a standard deviation of approximately 0.09cm, with 95% confidence interval (95%CI) and accuracy of 0.01cm relative to the true mean cecal appendix diameter. Therefore, the estimated sample size required for this study amounted to 188 patients. The final sample comprised 196 patients. Upper and lower reference limits for normal cecal appendix diameter for age were also inferred. Therefore, sample size was determined according to age group.

### Statistical analysis

Data were collected via analysis of images stored in Picture Archiving and Communication System (SBS PACS) and the search limited to US reports containing the term “normal cecal appendix”. The search was carried out using the BI-Reports system (Busca Laudos, 2019, São Paulo, SP, Brazil). Normal cecal appendix diameter measurements reported in children submitted to US examination were extracted. Child age and cross-sectional cecal appendix diameter were expressed as summary measures (means, standard deviations, median, minimum and maximum values). Child sex was described using absolute and relative frequencies. The estimated 95%CI for normal cecal appendix diameter was provided. Finally, maximum cecal appendix diameter was analyzed by age and compared using analysis of variance (ANOVA) followed by multiple Bonferroni comparisons.

## RESULTS

**Figure 3:**
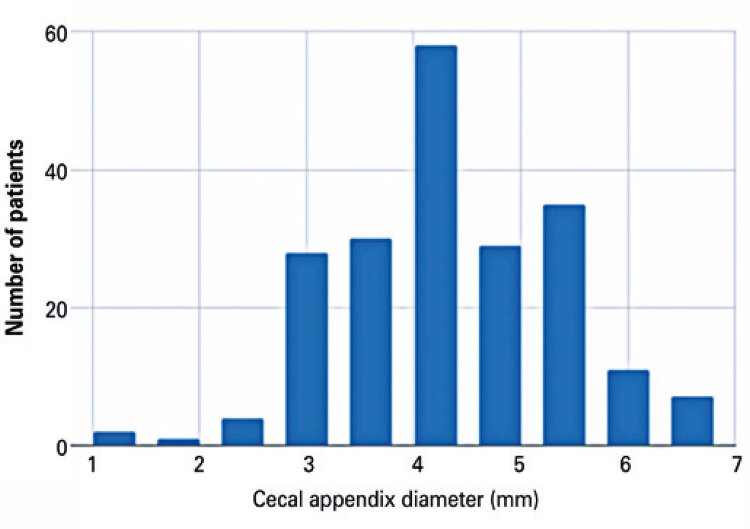
Distribution of normal cecal appendix diameter

The sample comprised 196 patients aged 0 to 15 years (mean age, 8.5 years). Sonographic measurements of the anteroposterior diameter of the cecal appendix resulted in a mean diameter of 4.14mm (standard deviation: 0.93mm; 95%CI: 3.86-4.14). The largest diameter detected was 6.2mm and the smallest 1.7mm (Figure 3).

**Figure 4:**
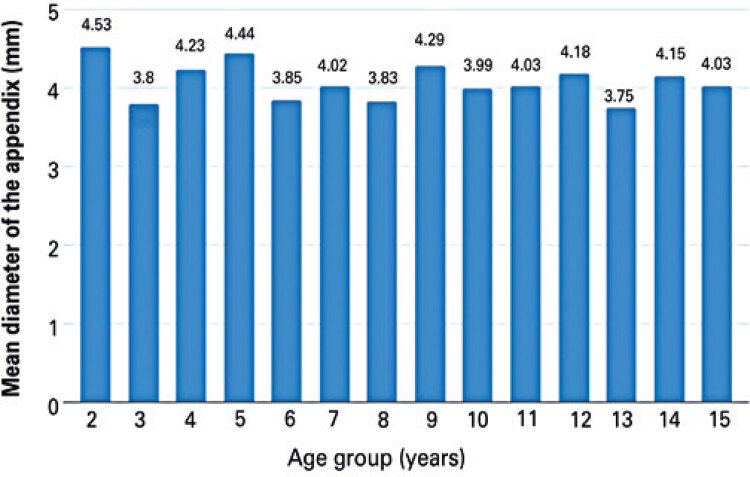
Mean cecal appendix diameter according to age

**Figure 5:**
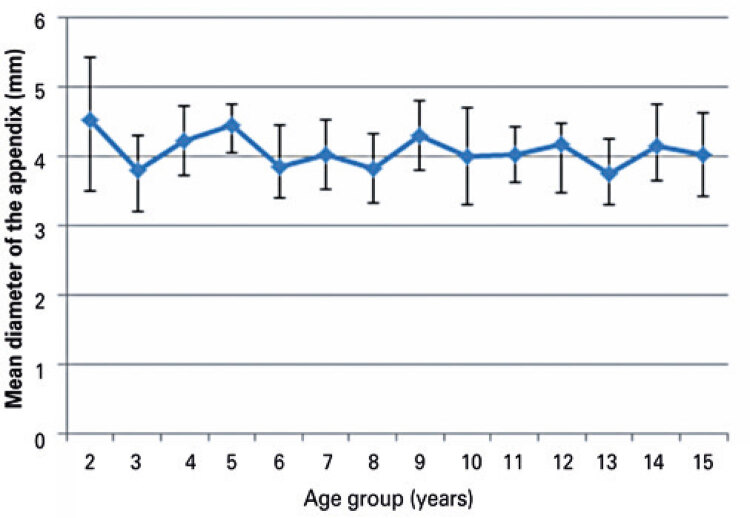
Mean cecal appendix diameter according to age

Cecal appendix diameter values were stratified by extremes of age (<5 or 15 years). The mean cecal appendix diameter in children aged <5 or 14 to 15 years was 4.2mm and 4.1mm respectively. Cecal appendix diameter did not differ significantly between age groups (Figures 4 and 5).

**Figure 6:**
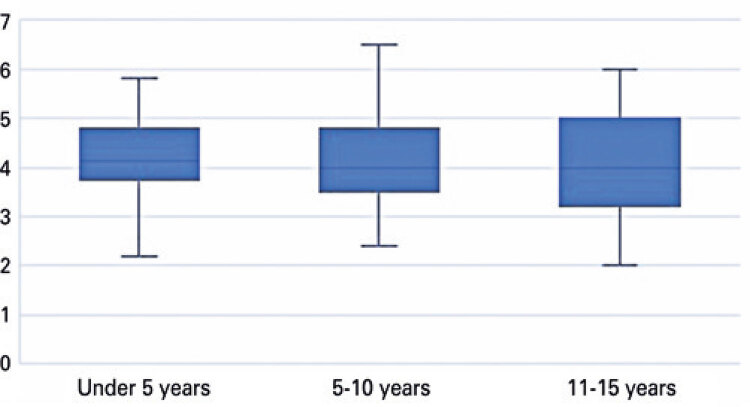
Boxplot displaying cecal appendix diameter according to age group

Cecal appendix diameter values were stratified by age group (<5, to 10 years and 11 to 15 years). The mean cecal appendix diameter in patients aged<5 years (56) was 4.2mm compared to 4.07mm in patients aged 5 to 10 years (68) and 4.08mm in patients aged 11 to 15 years (70). Cecal appendix diameter did not differ significantly between age groups (Figures 4, 5 and 6).

## DISCUSSION

Different from other studies, normal cecal appendix diameter was stratified by age and compared between age groups in this study. In other studies, comparisons were made within age groups (*e.g*., 0 to 15 years). Intergroup comparisons in this study enabled a more detailed analysis and failed to reveal significant differences between age groups.

Cecal appendix diameter is one of the most important parameters for US diagnosis of appendicitis.^(2)^ Therefore, reference ranges for cecal appendix diameter are a valuable resource in clinical practice, especially in pediatric populations.^(10)^

This study revealed a mean cecal appendix diameter of 4.1mm±0.9mm in children aged between 0 and 15 years. This finding is in keeping with the few similar studies published to date. Wiersma et al. reported a mean diameter of 3.9mm±0.8mm in children aged 2 to 15 years,^(11)^ whereas Ozel et al. described a mean diameter of 4.2mm±0.9mm in children aged 2 to 16 years.^(12)^

Age-stratified analysis revealed mean cecal appendix diameter values of 4.2mm and 4.1mm in children aged 0 to 4 and 14 to 15 respectively, with no significant differences between age groups. Similar results have been reported in prior studies, which failed to reveal statistically significant correlations between cecal appendix diameter and age or significant increase in cecal appendix diameter with age.^(13,14)^

Most studies do not recommend age cutoffs for maximum cecal appendix diameter in US diagnosis of appendicitis.^(15,16)^

On top of allowing detailed understanding of abdominal anatomy by virtual dissection, US complies with the ALARA (As Low As Reasonable Achievable) principle,^(17)^ which expresses concern regarding diagnostic imaging and sets standards for use of lowest amounts of radiation. This principle supports the applicability of ultrasonography as a non-ionizing imaging modality that should have priority for abdominal assessment in urgent cases. It is also a strategic tool for cecal appendix imaging in all age groups and particularly in pediatric patients. Sonographic imaging assessment may benefit all patients and has significant clinical impacts, since it prevents unnecessary exposure to and encourages the rational use of radiation.

In this context, the extreme importance of objective US parameters to guide the use of US, such as the normal appearance and dimensions of different organs, must be emphasized. In all areas of medicine, US may help beginners as well as specialized professionals to understand the morphofunctional correlation among different organs.

Ultrasonography should be the imaging modality of choice for initial assessment in cases suspected of acute appendicitis. The establishment of reference ranges for normal cecal appendix diameter in pediatric patients plays a fundamental role, since this condition is a major cause of acute abdomen in children. It may also facilitate appropriate communication between medical team members, avoiding delays in diagnosis as well as complications.

Studies aimed at establishing US scores for the diagnosis of acute appendicitis are currently underway in several centers worldwide. Hence, knowledge of descriptions of normal appendicular morphology is vital.

This study was carried out with Brazilian children and is consistent with the few international studies with similar scope published so far, in which similar findings have been reported.

The operator-dependent nature of US is a major limitation of this study, since this may have directly affected the technique employed, as well as results.

## CONCLUSION

This study revealed a mean value of normal cecal appendix diameter of 4.1mm±0.9mm. The findings suggest this value can be generalized to pediatric population overall, since normal cecal appendix diameter did not differ significantly according to age in this sample.

## References

[1] D’Agostino J. Common abdominal emergencies in children. Emerg Med Clin North Am. 2002;20(1):139-53. Review. 10.1016/s0733-8627(03)00055-5 11826631

[2] Botter LA, Oliveira GR, Farias JL, Mauramo A, Garcia RG, Queiros MR, et al. Ultrasonography in the diagnosis of acute appendicitis. einstein (São Paulo). 2005;3(3):185-9.

[3] Puylaert JB. Acute appendicitis: US evaluation using graded compression. Radiology. 1986;158(2):355-60. 10.1148/radiology.158.2.2934762 2934762

[4] Rettenbacher T, Hollerweger A, Macheiner P, Gritzmann N, Daniaux M, Schwamberger K, et al. Ovoid shape of the vermiform appendix: a criterion to exclude acute appendicitis--evaluation with US. Radiology. 2003;226(1):95-100. 10.1148/radiol.2261011496 12511674

[5] Konuş OL, Ozdemir A, Akkaya A, Erbaş G, Celik H, Işik S. Normal liver, spleen, and kidney dimensions in neonates, infants, and children: evaluation with sonography. AJR Am J Roentgenol. 1998;171(6):1693-8. 10.2214/ajr.171.6.9843315 9843315

[6] Binkovitz LA, Unsdorfer KM, Thapa P, Kolbe AB, Hull NC, Zingula SN, et al. Pediatric appendiceal ultrasound: accuracy, determinacy and clinical outcomes. Pediatr Radiol. 2015;45(13):1934-44. 10.1007/s00247-015-3432-7 26280637

[7] Mittal MK, Dayan PS, Macias CG, Bachur RG, Bennett J, Dudley NC, Bajaj L, Sinclair K, Stevenson MD, Kharbanda AB; Pediatric Emergency Medicine Collaborative Research Committee of the American Academy of Pediatrics. Performance of ultrasound in the diagnosis of appendicitis in children in a multicenter cohort. Acad Emerg Med. 2013;20(7):697-702. 10.1111/acem.12161 PMC5562364 23859583

[8] Trout AT, Sanchez R, Ladino-Torres MF. Reevaluating the sonographic criteria for acute appendicitis in children: a review of the literature and a retrospective analysis of 246 cases. Acad Radiol. 2012;19(11):1382-94. Review. 10.1016/j.acra.2012.06.014 22947273

[9] Fallon SC, Orth RC, Guillerman RP, Munden MM, Zhang W, Elder SC, et al. Development and validation of an ultrasound scoring system for children with suspected acute appendicitis. Pediatr Radiol. 2015;45(13):1945-52. 10.1007/s00247-015-3443-4 26280638

[10] Prendergast PM, Poonai N, Lynch T, McKillop S, Lim R. Acute appendicitis: investigating an optimal outer appendiceal diameter cut-point in a pediatric population. J Emerg Med. 2014;46(2):157-64. 10.1016/j.jemermed.2013.08.027 24113477

[11] Wiersma F, Srámek A, Holscher HC. US features of the normal appendix and surrounding area in children. Radiology. 2005;235(3):1018-22. 10.1148/radiol.2353040086 15914481

[12] Ozel A, Orhan UP, Akdana B, Disli C, Erturk SM, Basak M, et al. Sonographic appearance of the normal appendix in children. J Clin Ultrasound. 2011; 39(4):183-6. 10.1002/jcu.20807 21425275

[13] Coyne SM, Zhang B, Trout AT. Does appendiceal diameter change with age? A sonographic study. AJR Am J Roentgenol. 2014;203(5):1120-6. 10.2214/AJR.13.12205 25341153

[14] Searle AR, Ismail KA, Macgregor D, Hutson JM. Changes in the length and diameter of the normal appendix throughout childhood. J Pediatr Surg. 2013;48(7):1535-9. 10.1016/j.jpedsurg.2013.02.035 23895968

[15] Je BK, Kim SB, Lee SH, Lee KY, Cha SH. Diagnostic value of maximal-outer-diameter and maximal-mural-thickness in use of ultrasound for acute appendicitis in children. World J Gastroenterol. 2009;15(23):2900-3. 10.3748/wjg.15.2900 PMC2699009 19533813

[16] Kelly BS, Bollard SM, Weir A, O’Brien C, Mullen D, Kerin M, et al. Improving diagnostic accuracy in clinically ambiguous paediatric appendicitis: a retrospective review of ultrasound and pathology findings with focus on the non-visualised appendix. Br J Radiol. 2019;92(1093):20180585. 10.1259/bjr.20180585 PMC6435085 30102564

[17] Oestreich AE. RSNA centennial article: ALARA 1912: “As low a dose as possible” a century ago. Radiographics. 2014;34(5):1457-60. 10.1148/rg.345130136 25208291

